# Curcumins-Rich Curry Diet and Pulmonary Function in Asian Older Adults

**DOI:** 10.1371/journal.pone.0051753

**Published:** 2012-12-26

**Authors:** Tze Pin Ng, Mathew Niti, Keng Bee Yap, Wan Cheng Tan

**Affiliations:** 1 Gerontological Research Programme, Yong Loo Lin School of Medicine, National University of Singapore, Singapore, Singapore; 2 Department of Psychological Medicine, Yong Loo Lin School of Medicine, National University of Singapore, Singapore, Singapore; 3 Health Service Research and Evaluation, Ministry of Health, Singapore, Singapore; 4 Alexandra Hospital, Singapore, Singapore; 5 University of British Columbia James Hogg Research Centre, Heart Lung Institute, St Paul's Hospital, Vancouver, Canada; Children's Hospital Los Angeles, United States of America

## Abstract

**Background:**

Research on the effects of dietary nutrients on respiratory health in human populations have not investigated curcumin, a potent anti-oxidant and anti-inflammatory compound present principally in turmeric used in large amounts in Asian curry meals.

**Objectives:**

To examine the association of curry intake with pulmonary function among smokers and non-smokers.

**Design:**

The frequency of curry intake, respiratory risk factors and spirometry were measured in a population-based study of 2,478 Chinese older adults aged 55 and above in the Singapore Longitudinal Ageing Studies.

**Results:**

Curry intake (at least once monthly) was significantly associated with better FEV_1_ (b = 0.045±0.018, p = 0.011) and FEV_1_/FVC (b = 1.14±0.52, p = 0.029) in multivariate analyses that controlled simultaneously for gender, age, height, height-squared, smoking, occupational exposure and asthma/COPD history and other dietary or supplementary intakes. Increasing levels of curry intake (‘never or rarely’, ‘occasional’, ‘often’, ‘very often’) were associated with higher mean adjusted FEV_1_ (p for linear trend = 0.001) and FEV_1_/FVC% (p for linear trend = 0.048). Significant effect modifications were observed for FEV_1_ (curry* smoking interaction, p = 0.028) and FEV_1_/FVC% (curry*smoking interaction, p = 0.05). There were significantly larger differences in FEV_1_ and FEV_1_/FVC% between curry intake and non-curry intake especially among current and past smokers. The mean adjusted FEV_1_ associated with curry intake was 9.2% higher among current smokers, 10.3% higher among past smokers, and 1.5% higher among non-smokers.

**Conclusion:**

The possible role of curcumins in protecting the pulmonary function of smokers should be investigated in further clinical studies.

## Introduction

Curcumin (1,7-bis-(-4-hydroxy-3-methoxiphenyl)-1,6-heptadiene-2,5-dione) and curcuminoids (demethoxycurcumin, bisdemethoxycurcumin, and cyclocurcumin) is known to possess potent anti-oxidant and anti-inflammatory actions [1]–[4], but has never been considered as potential agents for respiratory health. Curcumin compounds (curcumins) are isolated from turmeric, an Indian yellow spice prepared from the dried rhizome of *Curcuma longa*, a member of the ginger family (*Zingiberaceae*), and used in large amounts as a food flavoring for curry meals in Asian populations. Turmeric has long been used traditionally for the treatment of coryza, indigestion, gallstone, hepatic disorders and rheumatism, and to promote healing of cuts and wounds.

A more potent antioxidant than vitamin E, curcumin inhibits in vitro lipid peroxidation and scavenges free oxygen and NO-based radicals, thus preventing oxidative damage of DNA. [5], [6] It also inhibits lipoxygenase and cyclooxygenase-2, [7] enzymes that are responsible for the synthesis of the pro-inflammatory leukotrienes, prostaglandins and thromboxanes, [8] and suppresses inducible nitric oxide synthase in activated macrophages. [9] Interestingly, curcumins demonstrated pulmonary protective effects against paraquat toxicity, [10] and attenuates elastase- and cigarette smoke-induced pulmonary emphysema in mice. [11] Currently, curcumins are being investigated as candidate compounds for the treatment of colonic polyps and cancer, Crohn's disease and dementia. [12] The possible protective effect of dietary curcumins in reducing the deleterious pulmonary effects of tobacco smoke among smokers has not been investigated.

Among the elderly, greater exposure to oxidative stress and lower dietary intake of antioxidants and micronutrients in foods may render them particularly vulnerable to lung damage, increasing the risk of chronic obstructive pulmonary disease (COPD). A growing body of evidence [13], [14] suggests that micronutrients including antioxidant vitamins A, C and E and selenium [14]–[26], 1,25-dihydroxy vitamin D [27]–[30] and omega-3 polyunsaturated fatty acids (n-3 PUFA) [31]–[34] and may play important roles in protecting the lungs from the effects of oxidative stress and chronic inflammation, especially from smoking. Curcumins have not been shown in any study to protect against the risk of smoking-associated obstructive pulmonary disease, but is a major source of dietary antioxidants in Asian diets, almost all from turmeric in curries.

In this study, we investigated the association of a turmeric (curcumins)-rich curry dietary intake with pulmonary function in a population sample of Chinese older adults. Because it was possible that curcumin intake may be correlated with the intake of other micronutrients and anti-oxidants including vitamins A, C, E and D and omega-3 PUFA, we also determined the pulmonary effect of curcumins independently of the intakes of these micronutrients in multivariate analyses. We tested the hypothesis that the anti-oxidant and anti-inflammatory effect of curcumins in curry may be evident in protecting against the pulmonary damage caused by smoking by investigating the effect of curry intake on pulmonary function of smokers and nonsmokers.

## Methods

### Study subjects

The study sample was drawn from participants in the Singapore Longitudinal Ageing Studies (SLAS), an observational cohort study of ageing and health among community-dwelling older persons. From September 2003 to December 2004, participants aged 55 and above were recruited by door-to-door census (N = 3894) from the whole population residing in five districts in South East Region, excluding those who were too severely incapacitated physically or mentally to give informed consent or participate. A total of 2804 residents participated in the study (response rate 78%). The study was approved by the National University of Singapore Institutional Review Board (NUS-IRB 04-140. After providing informed consent, the participants underwent extensive interviews and examinations that included measurements of pulmonary function. In this study, we obtained the data of 2608 Chinese respondents, and excluded in the analyses 81 respondents who did not perform spirometry, 46 with technically unsatisfactory spirometric performance and 3 with other missing data. Complete spirometric data was analyzed for 2478 respondents.

### Spirometry

Ventilatory function testing was performed using a portable, battery operated, ultrasound transit-time based spirometer (Easy-One; Model 2001 Diagnostic Spirometer, NDD Medical Technologies, Zurich, Switzerland). Forced expiratory maneuvers were performed with the respondent seated according to American Thoracic Society (ATS) recommendations on standardization of procedures^31^: at least three technically acceptable maneuvers, with the two best forced vital capacity (FVC) and forced expiratory volume in the first second (FEV_1_), reproducible to within 5% or 200 mL. The largest FEV_1_ and the largest FVC on any of the acceptable tests were used. Height and weight was measured with a portable Seca stadiometer (Model 708 1314004, Vogel & Hake Hamburg, Germany).

### Questionnaire

Reported frequency of usual intake of curry in meals were quantified as ‘never or rarely’ (never or less often than once in 6 months), ‘occasional’ (once in 6 months or more but less than once a month) and ‘often’ (once a month or more but less than once a week), and ‘very often’ (once a week or more, or daily). Interviewers distinguished other spicy foods such as chilly, coriander, tamarind, cinnamon, fenugreek, aniseed, cloves and others if they did not contain turmeric. Curry rich in turmeric was distinguished as those that clearly imparted a rich yellow color to the food.

We determined the intakes of supplements by asking participants the frequencies with which they regularly consumed vitamins A, C, E or vitamin D, omega-3 PUFA (alpha-linolenic acid, ALA, docosa hexaenoic acid, DHA, eicosa pentaenoic acid, EPA) and selenium: (1) never or rarely; (2) less than once a month; (3) more than once a month but less than 1 time a week; (4) more than once a week but not daily; (5) always (daily). The distributions were markedly bimodal, with 94% of the responses for ‘never or rarely’ or ‘daily’. Hence, the responses were dichotomized by daily intake of supplements (yes/no). There were no reports of any intake of curcumin supplements.

The participants were also asked in a brief semi-quantitative food frequency questionnaire whether they drank or ate ‘a lot of’ milk products (at least one serving everyday); ‘a lot of’ fruits or vegetables (at least one serving everyday); and ‘a lot of’ fish (more than 3 times a week).

Other data included age, gender, housing types (an established surrogate measure of socio-economic and income status), smoking (past or current smoker), past occupational exposure to dust or fumes, and reported past medical history of an asthma or COPD.

### Statistical analysis

The associations between levels of curry intake (primary independent variable of interest) and FEV_1_, FVC or FEV_1_/FVC (dependent variables) were determined using multiple linear regression. The regression models included *a priori* potential confounding co-variables which are known risk factors of pulmonary impairment established in the literature, and significant variables identified from initial univariate analyses (p<0.05). The primary confounding variables in all adjustment models for FEV_1_, FVC and FEV_1_/FVC% included appropriately gender, age (single years), height (cm), smoking status (non-smokers, past smoker, current smoker, less than 20 cigarettes per day, 20 or more cigarettes per day), past occupational history and reported past or recent history of asthma, and additionally a significant height-squared term, where appropriate. Body mass index, dietary and supplement variables (intakes of fruits or vegetables, fish, milk or dairy products, antioxidant vitamins A, C or E supplements, vitamin D supplement, omega supplement, selenium supplement) which were possible nutritional co-variables of curry intake, were identified from initial base models and significant variables (p<0.05) were added in sequential models for further adjustments of the coefficient estimates of association between curry intake and pulmonary variables.

Tests of linear trends in adjusted mean values of FEV_1,_ FVC and FEV1/FVC% across four ordinal categories of curry consumption were derived from estimated marginal mean values from ANCOVA in general linear model. Finally, we tested for significant interaction between curry intake (at least once a month versus less than once a month) and smoking status (non-smoker, past smoker and current smoker). All statistical tests were two-sided, and statistical significance was determined by *p*<0.05. Statistical analyses were performed using SPSS statistical software version 16.0 (SPSS Inc, Chicago Il).

## Results

The mean age of the participants was 66 years. (Table 1) Almost 10% of the participants reported consuming curry at least once a week, and 25% reported consuming curry at least once a month. The frequencies of reported daily intake of supplements were about 18% for vitamins A,C, E and D, 6.5% for omega-3 fatty acids, and 2.2% for selenium, with almost all of the remaining individuals reporting no consumption at all. A majority reported consuming at least one serving of fruits or vegetables daily, but about half consumed milk products daily or fish more than 3 times a week. The spearman correlations of curry intake with other dietary or supplementary intakes were 0.065 (0.001) for daily vitamin A,C or E supplement intake, 0.058 (p = 0.008) for vitamin D supplement, 0.058 (p = 0.004) for daily omega-3 PUFA supplement intake, 0.032 (p = 0.11) for selenium supplement, 0.067 (p = 0.001) for fish intake 3 or more times a week. −0.019 (p = 0.34) for daily fruits or vegetables intake, and −0.030 (p = 0.14) for daily milk and daily intake.

**Table 1:** Characteristics of study participants (Singapore Longitudinal Ageing Studies).

		Total N		Very often		Often		Occasional		Rarely or never		
		Mean±SD		% or mean	(N) or ±SD	% or mean	(N) or ±SD	% or mean	(N) or ±SD	% or mean	(N) or ±SD	
Whole sample				228		401		1490		359		
Age (years), mean (SD)		65.9	±7.6	64.9	±7.0	65.1	±7.0	66.1	±7.8	66.5	±7.6	0.012
Gender	Male	914		41.7	(95)	43.1	(173)	35.7	(532)	31.8	(114)	0.003
Housing status	1–3 room HDB	712		24.1	(55)	23.2	(93)	29.3	(436)	35.7	(138)	0.001
	4–5 room HDB	1039		39.9	(91)	39.2	(157)	42.6	(634)	43.7	(157)	
	Higher end public or private	727		36.0	(82)	37.7	(151)	28.2	(420)	20.6	(74)	
Smoking	Non-Smoker	2066		86.4	197	82.5	331	83.2	1240	83.0	298	0.62
	Ex-Smoker<20 cigarettes daily	161		5.3	12	7.7	31	6.1	91	7.5	27	
	Ex-Smoker ≥20 cigarettes daily	94		3.1	7	3.2	13	4.0	60	3.9	14	
	Current Smoker<20 cigarettes daily	128		4.4	10	4.7	19	5.3	79	5.6	20	
	Current Smoker≥20 cigarettes daily	29		0.9	2	1.7	7	1.3	20	0.0	0	
Reported asthma or COPD	Yes	76		6.6	15	3.2	13	2.6	39	2.5	9	0.012
Past occupational exposure	Yes	122		5.7	13	4.0	16	5.0	74	5.3	19	0.76
Fruits or vegetable consumption	At least one serving daily	2274		88.2	201	93.0	373	91.8	1368	92.5	332	0.17
Vitamin A supplement	Daily	113		7.0	16	6.0	24	4.0	60	3.6	13	0.08
Vitamin C supplement	Daily	286		11.8	27	13.7	55	11.5	172	8.9	32	0.23
Vitamin E supplement	Daily	207		10.5	24	10.7	43	8.3	124	4.5	16	0.009
Vitamins A, C or E supplements	Daily	462		19.7	45	23.2	93	18.7	278	12.8	46	0.003
Milk or dairy products consumption	At least one serving daily	1254		53.1	121	46.9	188	50.1	746	55.4	199	0.10
Vitamin D supplement daily	Daily	443		19.7	45	21.2	85	17.7	264	13.6	49	0.047
Fish consumption	More than 3 times per week to daily	1237		47.8	109	57.9	232	50.1	747	41.5	149	0.001
Omega supplement	Daily	160		9.2	21	8.7	35	5.8	87	4.7	17	0.029
Selenium supplement	Daily	54		3.1	7	3.0	12	1.9	29	1.7	6	0.41
Height (metre)	Mean ±SD	1.58	±0.08	1.59	±0.07	1.59	±0.08	1.58	±0.08	1.57	±0.08	0.001
Body mass index, Kg/m^2^	Mean ±SD	23.6	±3.6	23.8	±3.6	23.9	±3.6	23.6	±3.6	23.3	±3.4	0.12
Forced expiratory volume,1s,, litres	Mean ±SD	1.82	0.54	1.91	0.55	1.93	0.51	1.81	0.55	1.69	0.50	0.001
Forced vital capacity, litres	Mean ±SD	2.44	0.71	2.51	0.73	2.55	0.67	2.43	0.73	2.28	0.65	0.001
FEV_1_/FVC, %,	Mean ±SD	75.4	11.5	77.1	11.1	76.4	10.0	75.0	11.8	74.7	12.1	0.011

Very often (≥ once a week or daily); Often (≥ once a month to < once a week; Occasional (once in 6 months to <once a month); Rarely or never (<once in 6 months).

Table 2 shows in the base model the expected significant independent associations of gender, age, height, height-squared, housing status, smoking, occupational exposure, and asthma/COPD history with FEV_1_ , FVC and FEV_1_/FVC% (R^2^ = 0.51). When added to the base model, curry intake (B = 0.049±0.018, p = 0.005) showed an independent positive associations with FEV_1_ (Model 1). When other dietary and supplementary intakes were added and analyzed simultaneously in the model, curry intake remained independently associated with FEV_1_.

**Table 2:** Multiple regression analysis of relationships of dietary and supplemental micronutrient consumption with forced expiratory volume in one second (FEV1), forced vital capacity (FVC) and FEV_1_/FVC.

	FEV_1_, litres				FVC, litres				FEV1/FVC, %			
	b	SE	t	p	b	SE	t	p	b	SE	t	p
Base model												
Intercept	10.627	2.273	4.676	<.001	13.451	3.214	4.186	<.001	37.323	67.088	.556	.58
Male gender*	0.321	0.023	13.863	<.001	.371	.033	11.321	<.001	1.405	.684	2.054	.040
Age, single year	−.025	.001	−23.081	<.001	−.027	.002	−17.541	<.001	−.189	.032	−6.003	<.001
Height, cm,*	−11.57	2.850	−4.060	<.001	−15.02	4.030	−3.728	<.001	64.132	84.134	.762	.45
Height-squared	4.418	.896	4.930	<.001	5.769	1.267	4.552	<.001	−20.25	26.457	−.766	.44
Body mass index	.001	.002	−.215	.83	−.004	.003	−1.450	.147	.082	.063	1.299	.194
Low end public housing*	−.080	.020	−3.919	<.001	.000	.029	.013	.99	−3.112	.600	−5.185	<.001
Mid-range public housing*	−.054	.018	−2.943	.003	−.010	.026	−.382	.70	−1.991	.543	−3.664	<.001
Current smoker, ≥20 cigarettes daily	−.153	.072	−2.131	.033	.024	.102	.236	.81	−7.054	2.120	−3.327	<.001
Current smoker, <20 cigarettes daily	−.152	.036	−4.198	<.001	−.009	.051	−.179	.86	−5.120	1.066	−4.803	<.001
Past smoker, ≥20 cigarettes daily	−.053	.042	−1.264	.21	−.049	.059	−.827	.41	−1.105	1.234	−.896	.37
Past smoker, <20 cigarettes daily	−.033	.033	−1.014	.31	.006	.046	.125	.90	−1.107	.965	−1.147	.25
Past occupational exposure	−.027	.036	−.763	.45	.037	.051	.737	.46	−2.346	1.061	−2.210	.027
Reported asthma or COPD	−.321	.044	−7.251	<.001	−.198	.063	−3.164	.002	−7.914	1.309	−6.047	<.001
Curry at least once a month												
Adjusted for significant variables in base model	.049	.018	2.787	.005	.027	.025	1.097	.27	1.265	.522	2.424	.015
Adjusted further for diet and supplements	.045	.018	2.536	.011	.025	.025	.99	.32	1.140	.521	2.187	.029

* Referenced to: female gender, higher end public or private housing, never smoker, no occupational exposure, and less frequent consumptions of fruits and vegetables, milk, fish and curry.

**Figure 1:**
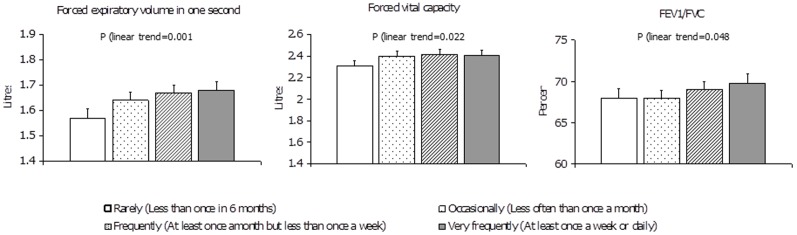
Adjusted mean forced expiratory volume in one second (FEV1), forced vital capacity (FVC) and FEV1/FVC% by levels of curry intake. Footnote: Bars denote standard errors. * P<0.05, ** p<0.01, *** P<0.001 FEV1 and FVC: Estimated marginal means adjusted for gender, age, height, height-squared, housing status, smoking, and history of asthma/COPD. FEV1/FVC: Estimated marginal means adjusted for gender, age, housing status, smoking, and history of asthma/COPD, and occupational exposure.

There was a linear trend increase in FEV_1_ associated with greater frequency of curry intake, controlling for gender, age, height, smoking and other covariables. The test for trend across the frequency categories was significant (p = 0.001) (Figure 1). Compared to participants who rarely or never consumed curry (adjusted mean FEV_1_ = 1.57 litres), participants who consumed curry occasionally (adjusted mean FEV_1_ = 1.64 litres), or often (adjusted mean FEV1 = 1.67 litres), or very often (at least weekly to daily, adjusted mean FEV_1_ = 1.68 litres) showed a 4.3%, 6.7% and 6.3% increase in mean FEV_1_ respectively. Similar trends were observed for FVC and FEV1/FVC%.

**Figure 2:**
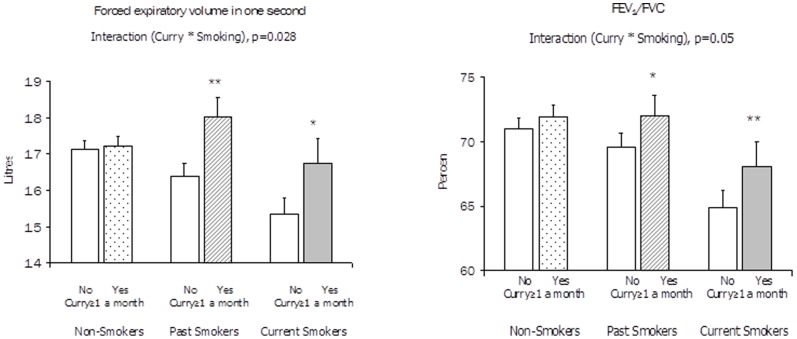
Adjusted mean forced expiratory volume in one second (FEV_1)_) and FEV_1_/FVC by curry consumption status among non-smokers, past smoker and current smokers. Footnote: Bars denote standard errors. * P<0.05, ** p<0.01, *** P<0.001 FEV1: Estimated marginal means adjusted for gender, age, height, height-squared, housing type, and asthma/COPD history. FEV1/FVC: Estimated marginal means adjusted for gender, age, housing type, asthma/COPD history and occupational exposure.

The association of curry intake (at least once a month) with FEV_1_ was found to vary significantly by smoking status (current, past, and non-smokers). The test of interaction was significant (p = 0.028). Curry consumption was associated with much greater differences in FEV_1_ among current smokers and past smokers than among non-smokers. Among current smokers, the adjusted mean FEV_1_ for non-curry intake was lowest at 1.53 litres; curry intake was associated with 9.2% higher adjusted mean FEV_1_. Among past smokers, the adjusted mean FEV_1_ for non-curry intake was 1.63 litres; curry intake more than once monthly was associated with 10.3% higher mean adjusted FEV_1_. Among non-smokers, the adjusted mean FEV1 for non-curry intake was highest at 1.71 litres, whereas the adjusted mean FEV1 for curry intake was only marginally 1.5% higher. Similar results were observed for FEV_1_/FVC%. See Figure 2.

We further analyzed differences in pulmonary function between curry intake (at least once a month) and non-curry intake among a small number of participants who reported a history of asthma or COPD (N = 76). We found consistent results of higher mean adjusted FEV_1_ (b = +0.335 ± SE = 0.104, p = 0.002) and FVC ((b = +0.324 ± SE = 0.143, p = 0.027) and FEV1/FVC% (b = +4.50 ± SE = 3.37, p = 0.18) associated with curry consumption as well.

## Discussion

In this population-based study of Chinese middle aged and older adults, we found that the a turmeric (curcumins)-rich curry diet was significantly associated with better pulmonary function, controlling for potential confounding by known risk factors for COPD. Since it was possible that curcumin intake may be correlated with the intake of other nutrients including vitamins A, C, E and D, selenium and omega-3 PUFA, a protective effect attributed to curcumins may actually reflect the effect of another correlated antioxidant or anti-inflammatory nutrient(s), or an interaction between dietary constituents. We hence controlled for the pulmonary effects of the intakes of other anti-oxidant/anti-inflammatory nutrients in multivariate analyses and found no evidence that they could explain the pulmonary effect associated with curry intake. Our results thus suggested that dietary curcumins intake in curry had a positive effect on pulmonary function independent of other anti-oxidant and anti-inflammatory micronutrients. Furthermore, the significant linear trends of pulmonary function levels associated with increasing frequencies of curry intake suggest a clear dose-effect relation.

We investigated the effect of curry intake on pulmonary function among smokers and found that smokers who consumed curry showed levels of FEV_1_ and FEV_1_/FVC that were substantially higher than smokers who did not consume curry. These levels of FEV_1_ and FEV_1_/FVC% among smokers who consumed curry were almost similar to the levels observed among non-smokers. Among non-smokers, the smaller differences in pulmonary function associated with curry intake were perhaps not surprising, given the high functioning level for their age and possible ceiling effects. These results suggest that the anti-oxidant and anti-inflammatory actions of curcumins in curry might be particularly effective in protecting against pulmonary damage caused by smoking.

Given that smokers are exposed to large concentrations of oxidants in cigarette smoke, [35] hypothetically a stronger association of anti-oxidants with pulmonary function in smokers is expected if anti-oxidants could prevent oxidative damage. So far, very few studies possessed sufficient power to detect a statistically significant interaction of antioxidant intake with smoking. To our knowledge, only one study that analyzed a large data set in the NHANES III [22] has reported a stronger correlation of vitamin C with FEV_1_ in current smokers. Our study was sufficiently powered to observe the modifying effect of dietary curcumins on pulmonary function impairment associated with smoking.

The strengths of the present study include its large sample size, and the selection of an older adult population who are vulnerable to the effects of oxidative injury and nutritional deficiency and were hence at increased risk of obstructive pulmonary disease. We controlled for a large number of known risk factors for COPD that were potentially confounding variables in multivariate analyses, and obtained robust results for their expected pulmonary effects. We also measured dietary and supplementary intakes of multiple other anti-oxidants and anti-inflammatory nutrients, because a protective effect attributed to one antioxidant or micronutrient may actually reflect the effect of another correlated dietary constituent, or an interaction between dietary constituents. Our analysis suggested that the pulmonary effect of dietary curcumin was independent of other antioxidants and anti-inflammatory micronutrients.

Some limitations in this cross-sectional study should be noted. Although we attempted to control for the effects of other anti-oxidant and anti-inflammatory nutrients in the diet and supplements, the semi-quantitative food frequency questionnaire we used were limited, and did not include total energy intake; a 24 hour dietary recall methodology is preferred but more expensive. However, our analyses of the pulmonary effects for individual dietary and supplementary intakes of other anti-oxidant and anti-inflammatory nutrients in the regression models showed in fact that daily supplementary vitamin A/C/E (b = 0.049±0.020, p = 0.015), dietary fish intake at least thrice weekly (b = 0.059±0.016, p = 0.001), and daily supplementary n3-PUFA (b = 0.073±0.032, p = 0.021), were individually associated with FEV_1_ in the same regression model (data not shown). It may be argued that with cross-sectional results, the observed associations may possibly be explained by dietary change resulting from poor pulmonary function. However, in patients with COPD, this is generally expected to result in reduced food intake and under-nutrition. Community-living older persons possessing varying levels of pulmonary function include a sub-population of individuals who have COPD but it is well known that they are generally unaware of its presence, and it seems very unlikely that they would accordingly increase their dietary intake especially for curry. Nevertheless, residual confounding from inadequate measurements and unknown variables may still be possible.

The results of this preliminary study support the hypothesis that dietary intake of curcumins in a turmeric-rich diet almost wholly in curry among Asians had a positive effect on pulmonary function. The possible role of curcumins in protecting the pulmonary function of smokers should be further investigated in clinical studies.
